# Programmable Porosity in Polymers via Microfluidic Templating and 3D Printing

**DOI:** 10.1002/smtd.71070

**Published:** 2026-09-27

**Authors:** Federico Serpe, Roberta Angelini, Andrea Barbetta, Vanessa Rosciardi

**Affiliations:** ^1^ Institute of Physical Chemistry Polish Academy of Sciences Warsaw Poland; ^2^ Department of Chemistry Sapienza University of Rome Rome Italy; ^3^ Institute for Complex Systems–National Research Council (ISC‐CNR) and Department of Physics Sapienza University of Rome Rome Italy

**Keywords:** emulsion templating, foam templating, liquid templating, microfluidics, polymeric functionally graded porous materials, porous materials

## Abstract

Polymeric functionally graded porous materials (pFGPMs) enable spatial control over transport and mechanical response in a continuous solid in which porosity is strategically placed rather than uniformly distributed. Early research on the production of pFGPMs has demonstrated that graded architectures can be induced as indirect outcomes of time‐evolving temperature, pressure, or concentration fields. Because these gradients emerge from process‐driven kinetics, reproducibility and spatiotemporal control remain limited. Fluid templating offers a more direct route: the transient structure of polymerizable/crosslinkable foams or high internal phase emulsions (HIPEs) can be transferred into a solid matrix while controlling compartment size, packing, and film stability during solidification. Microfluidics enhance fluid management by confining interfacial breakup to produce monodisperse bubbles or droplets whose size, volume fraction, and interconnectivity can be tuned independently, transforming liquid templates into programmable solid architectures. This review follows the evolution of microfluidic templating toward its integration with additive manufacturing, with an emphasis on platforms that embed in‐line bubble or droplet generation at the printhead. We conclude with an outlook on the opportunity to expand the palette of available materials and discuss the emerging role of data‐driven approaches in managing the coupled dynamics of formulation, breakup, and solidification, enabling truly programmable polymeric FGPMs.

## Graphical abstract

Fluid templating is a powerful way to produce porous polymers. Microfluidic technologies enable fine control over the dimension and packing of bubbles and droplets. Combined with additive manufacturing, microfluidic templating enables the creation of polymeric functionally graded porous materials in which both porosity and three‐dimensional geometry can be programmed on demand.

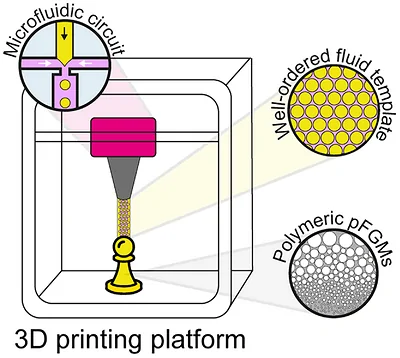

## 1 Introduction

Porous polymers derive their performance from architecture as much as from chemistry [1]. The mechanics, thermal response, and transport in these materials are governed by the topology of the void network (e.g., pore size, connectivity, and distribution) rather than solely by the intrinsic features of the polymer backbone [2, 3, 4]. Biological systems routinely use spatially‐organized heterogeneity to meet competing demands within a single structure [5, 6]. Indeed, gradients in porosity or microstructure can result in objects characterized by features that would otherwise be mutually exclusive (stiffness and compliance [7, 8], transfer efficiency and selectivity [9], surface area and stability) [10]. This logic motivates the growing interest in polymeric functionally graded porous materials (pFGPMs), in which porosity is intentionally varied along a designated direction to tune local behavior [11]. However, translating that logic into fabrication remains challenging. Canonical routes (e.g., mechanical frothing [12], sacrificial templating [13], physical [14] or chemical [15] blowing agents in polymer melts) readily introduce voids but struggle to organize them in the desired spatial arrangement. If present, gradients are typically the consequence of evolving fields (temperature [16], pressure [17], local composition [18]), which are difficult to stabilize or scale. Even additive manufacturing, despite its geometric freedom, largely scripts porosity at the macroscale, leaving the microscale to emerge from formulation and curing history [19]. As many applications depend on where porosity is placed [20], and not simply on its presence, the need for approaches that finely control voids arrangement becomes increasingly clear. What is missing from canonical routes is not the ability to generate pores, but the ability to organize them in space. Fluid templating comes in help by allowing the transient architecture of foams and high internal phase emulsions (HIPEs) to be transferred into a porous solid [21, 22]. Because the pore network is inherited from bubbles or droplets, controlling the templating dispersion ultimately controls the material. Yet, in bulk foaming and emulsification, broad size distributions and ageing processes (drainage, coarsening, coalescence) blur the direct correspondence between the fluid formulation and the final solid structure, making gradients difficult to prescribe and hierarchy challenging to preserve.

Microfluidic foaming and emulsification recover determinism by confining interfacial breakup to geometries where viscous and capillary forces dominate [23]. Under such confinement, compartment size and spacing are set by flow conditions and geometry rather than stochastic instabilities and gravity‐driven phenomena, allowing ordered packings to persist long enough to be locked in by gelation or polymerization [24]. Crucially, microfluidics converts fluid templating from a static precursor into a programmable process: bubble or droplet size, gas/liquid fraction, and temporal sequences can be varied on demand, making porosity a spatiotemporal design variable rather than an uncontrolled outcome. Integrating these capabilities with additive manufacturing shifts the paradigm from printing shapes that happen to be porous to directly writing porosity itself. When microbubble or microdroplet generation is embedded within the printhead, the templating dispersion can be modulated during deposition: pore size, density, and even local composition can be scripted along the toolpath with the same intentionality as the external geometry. Microfluidic‐assisted 3D printing thus unifies architectural freedom with microscopic precision, enabling the production of polymeric FGPMs whose microarchitecture is actively imparted rather than the secondary result of specific processing conditions.

In what follows, we establish the limitations of conventional routes to achieve graded porosity and motivate why deterministic control over templating dispersions is essential; we then distil the physics that make microfluidic foams and concentrated emulsions architecturally stable and tunable; finally, we survey fabrication platforms that embed microfluidic control into 3D printing platforms to produce polymeric FGPMs with programmable gradients, hierarchical porosity, and complex microgeometries. This progression, from classical methods to microfluidic templating and integrated manufacturing, maps the methodological evolution of the field and frames the opportunities at the interface of soft‐matter physics, interfacial engineering, and advanced manufacturing. Looking ahead, we conclude providing an outlook on how a broader materials palette and learning‐based, data‐driven process integration may further expand this landscape, offering a glimpse of what truly programmable porosity could become.

## 2 Background

### 2.1 pFGPMs: The First Approaches

Functionally graded porous materials reconcile otherwise conflicting requirements (e.g., stiffness/compliance [7, 8], permeability/selectivity [9], surface area/stability) by allowing local porosity to vary along a designated direction with smooth architectural changes [2]. In polymers, this strategy is especially powerful because the matrix chemistry can be tuned (e.g., through the modulation of crosslinking density, copolymer composition, and plasticization) without changing how voids are architected. The persistent difficulty has been to “write” those pore gradients with precision rather than letting them emerge from processing conditions. Gas foaming approaches are a clear example of process‐driven porosity generation. In such methods, gradients can be induced indirectly by establishing CO_2_ concentration fields within a polymer prior to depressurization, so that bubble nucleation and growth rates vary spatially (Figure 1A,i). To induce the emergence of graded porosity in thermoplastic polyurethane, for example, Zhu et al. harnessed staged saturation to program nonuniform gas uptake thereby obtaining well defined cellular gradients [25]. Analogous logic has been demonstrated in polystyrene and polycaprolactone, where Trofa et al. showed that time dependent boundary conditions during sorption generate persistent concentration profiles that translate into graded porosity upon foaming [26]; in polymethylmethacrylate (PMMA), Zhou et al. employed partial saturation under supercritical CO_2_ to create nonequilibrium gas distributions and, consequently, structured gradients [27]; While effective, these routes rely on tight control of pressure/temperature histories and diffusion distances, which complicates scale‐up and reproducibility. Crucially, these gradients are written indirectly: they depend on how local kinetics happen to unfold, rather than on a pre‐imposed architectural design. As a result, two samples produced under nominally identical macroscopic conditions can diverge at the microscopic scale, underscoring the difficulty of translating stimulus‐driven gradients into reproducible design tools.

**FIGURE 1:**
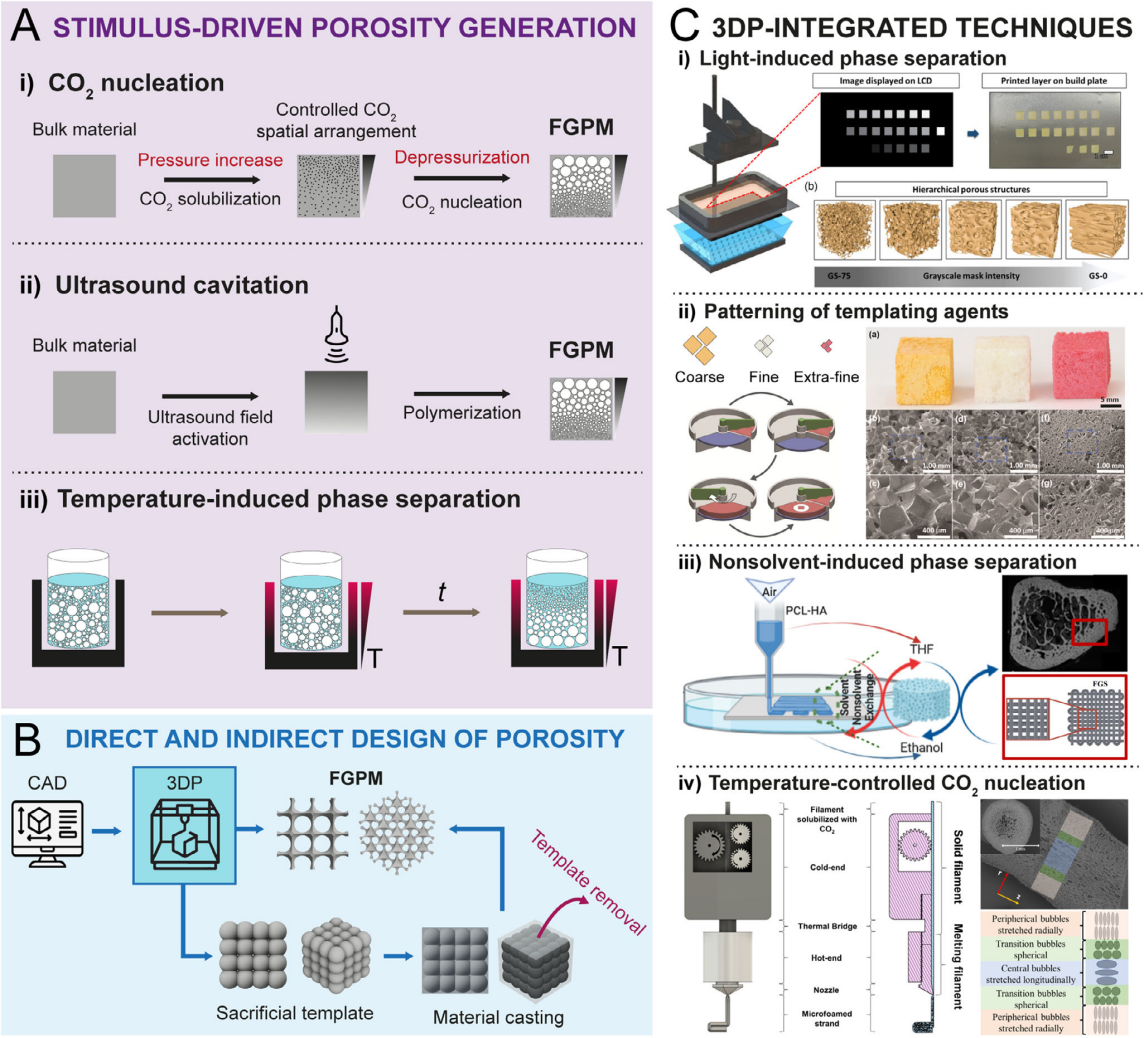
Summary of conventional strategies to produce polymeric functionally graded porous materials (pFGPMs). Most reported approaches rely on stimulus‐induced processes, evolution of external fields that influence phase‐separation, selective polymerization, and sacrificial use of solid templates. For clarity, the methods described in recent literature are broadly grouped into three main categories: (**A**) stimulus‐driven porosity generation, (**B**) direct and indirect 3D printing design of porosity, and (**C**) hybrid strategies that combine externally triggered porosity with the geometric freedom offered by 3D printing. (**C, i**) Greyscale Digital Light Processing (G‐DLP) enabled the modulation of the irradiation dose to trigger selective phase separation of photocurable inks to ultimately obtain functionally graded porous materials. Reproduced with permission under the CC‐BY licence [34] 2023, The Authors. Small Structures published by Wiley‐VCH GmbH. (**C, ii**) The combination of DLP and salt leaching enabled the fabrication of porous polymeric samples. By switching the ink containing salt grains from coarse to extra‐fine, pore size distribution can be controlled along the 3D construct. Reproduced with permission [35] 2017, The Royal Society of Chemistry. (**C, iii**) The integration of extrusion‐based 3D printing with the nonsolvent‐induced phase separation (NIPS) method enabled the fabrication of scaffold with multiscale porosity. Reproduced with permission under the CC‐BY licence [36] 2025, The Authors. Published by American Chemical Society. (**C, iv**) The generation of strong thermal gradients within extruded polymeric strand, combined with Fused Filament Fabrication (FFF), enabled the formation of micro‐foamed filaments with anisotropic bubble orientation. Reproduced with permission [37] 2024, American Chemical Society.

Other stimulus‐driven methods face analogous constraints. Torres‐Sánchez and co‐workers introduced low‐frequency ultrasound during foam formation (temporally coincident with the liquid system's polymerization) to perturb nucleation, drainage, and coarsening, producing spatially varying porosity within a single monolith (Figure 1A, ii) [28]; the outcome, however, depends on acoustic field heterogeneity and on the timing within the gelation/foaming window [29]. In polymeric gels, freeze‐drying is well known to generate porosity robustly, and gradients can be encoded upstream (radially by centrifugation [30] or by layer stacking before lyophilization [31]) but the final morphology is governed by solvent crystallization and drying fronts. Thermally induced phase separation can also be harnessed (Figure 1A, iii): Mannella et al. [32] and Tialiou et al. [33] imposed temperature gradients across polymeric solutions or thermoresponsive hydrogels to obtain pronounced pore gradients; here the architecture is set by demixing kinetics and quench rate, with limited knobs to tune hierarchy and interconnectivity independently.

Additive manufacturing expands geometric freedom, yet conventional implementations mainly program macroporosity, while microscale voids still arise indirectly. Hybrid strategies have begun to narrow this gap. Balakrishnan et al. coupled grayscale digital light projection (DLP) with polymerization‐induced phase separation, sweeping a single formulation from dense to porous within a layer (Figure 1C, i) [34]. Despite this work implements a powerful concept, pore sizes remain exclusively sub‐micrometric and constrained by photochemistry. Mu et al. blended sacrificial salt porogens with different granulometries into DLP resins to produce large, open pores after leaching [35]; here, connectivity is largely set by random packing, and the control over gradients is governed by the selection of the right grain size in each layer (Figure 1C, ii). In sacrificial‐template inversion, Montazerian et al. printed minimal‐surface scaffolds (via fused deposition modelling—FDM) and cast polydimethylsiloxane (PDMS) to obtain radially graded architectures [38]. The proposed approach is elegant but multi‐step and constrained by mould removability, which inevitably yields connected pores. Bilgili et al. integrated extrusion with non‐solvent induced phase separation to achieve dual‐scale porosity (Figure 1C, iii) [36]; while effective, this approach ties microstructure to solvent‐exchange kinetics and bath conditions. In the same period, Esposito et al. combined 3D printing with CO_2_ foaming by solubilizing CO_2_ into a polylactic acid (PLA) filament and foaming in situ during extrusion to produce orientation‐graded microcellular strands (Figure 1C, iv) [37]. In their work, they imposed high printing velocities to generate strong radial temperature gradients (a flow regime characterized by high Graetz numbers); in these conditions, bubbles elongate axially in the cooler core and radially in the hotter periphery, yielding a continuous double morphology that arises solely from the thermal‐fluidic conditions rather than changes in the formulation. Recently, Posabella et al. advanced CAD‐controlled pore gradients by generating spherical‐based graded architectures via an automated script and printing them with low‐force stereolithography [39]. Their study broadens the CAD design space but also underscores the limits of high‐resolution light‐based printing. For example, many architectures were nonprintable or mechanically fragile due to feature size and thin strut failures, others, instead, showed overcuring and restricted solvent access that produced unintended bulk regions, and, at porosities above 80%, printing inaccuracies and shrinkage dominated, reducing fidelity and widening model‐experiment discrepancies. Regarding vat photopolymerization techniques, Sengokmen‐Ozsoz and co‐workers proposed the integration of preformed liquid templates upstream of printing. They formulated MIPE/HIPE (medium and high internal phase emulsion) resins compatible with commercial SLA printers by incorporating tartrazine that suppresses scattering and introduces secondary porosity [40]. They reproduced multiscale trabecular architectures (reconstructed from micro‐CT scans), achieving ≈150 µm feature resolution with embedded micro‐ and macro‐porosity. While these systems offer excellent geometric fidelity, porosity remains fixed during printing unless different HIPEs/MIPEs or exposure‐dose modulation strategies are used, limiting their ability to produce spatial gradients.

The common thread that binds all the mentioned approaches is that porous gradients are outcomes of evolving fields (e.g., pressure, temperature, local concentration, acoustic intensity) rather than the result of deterministic control over the compartments that ultimately template pore size, throat geometry, and connectivity. Without control over bubbles, droplets, and thin films separating them, gradient profiles must be inferred from processing paths that are sensitive to the kinetics of the evolving system and the geometry of the setup. This limitation motivates the shift toward fluid templating, in which the transient architecture of foams and HIPEs is treated as the design variable itself. The next section examines how this templating logic unifies foams and HIPEs at the level of interfacial geometry and film‐scale physics, setting the stage for microfluidic approaches that allow deterministic control.

### 2.2 Emulsion and Liquid Foam Templating

Liquid foams and HIPEs offer two parallel routes toward the production of porous polymers based on the core principle of fluid templating: a transient biphasic fluid assembly defines the architecture, and solidification of the external phase permanently retains it. In both systems, the structure of the dispersed phase (i.e., gas bubbles in foams, oil/water droplets in HIPEs) dictates the topology of the void network [41, 42]; gelation or polymerization of the continuous phase transfers that topology to the solid [43, 44]. Because the solid inherits its morphology directly from a multiphase fluid, control over this architecture depends on managing the physics of the template itself, that is how bubbles and droplets are generated, how they pack, and how long that packed configuration can be maintained before destabilization. The design problem therefore reduces to controlling (i) the compartment size and monodispersity, (ii) the thickness and stability of the separating films, and (iii) the kinetics by which the structure remains ‘frozen’ before drainage, coarsening, or coalescence can erase order [45]. Figure 2 illustrates these templating strategies, showing that, despite their differences, they face common challenges associated with instability phenomena. In this section, we provide the essential background knowledge required to read this review. As the subject is broad and multifaceted, interested readers are encouraged to consult the foundational literature for a more comprehensive treatment [46].

**FIGURE 2:**
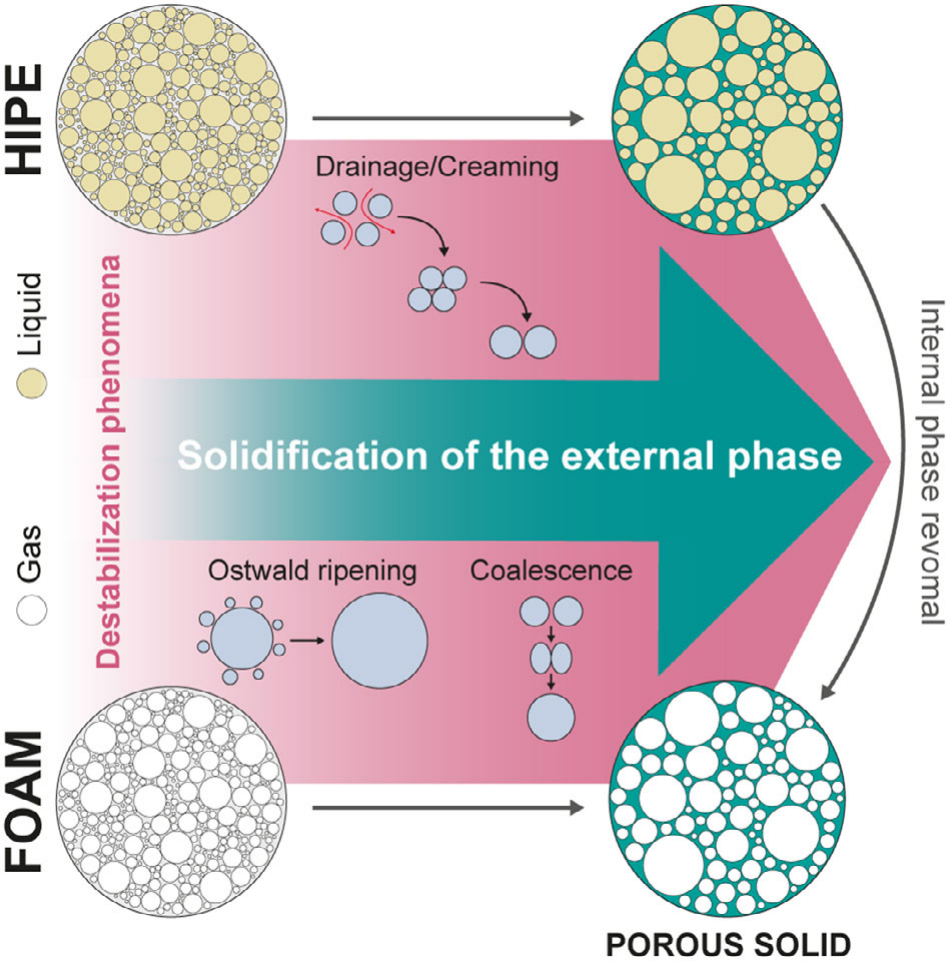
Conceptual representation of fluid templating as a competition between destabilization processes in multiphase fluids and solidification of the external phase. In high internal phase emulsions (HIPEs) and liquid foams, the dispersed architecture is initially defined in the liquid state but continuously evolves through destabilization phenomena (drainage/creaming, Ostwald ripening, and coalescence). Solidification of the external (continuous) phase arrests this evolution and transfers the transient liquid architecture into a porous solid. The final pore size, distribution, and connectivity therefore depend on the relative timescales of destabilization and solidification. The schematic highlights how careful synchronization of fluid templating and solidification is central for translating foams and emulsions into porous materials with controlled geometries.

In liquid foams, thin films evolve under capillary pressure gradients and gravity‐driven drainage, while coarsening proceeds via gas diffusion from high‐curvature to low‐curvature bubbles (Ostwald ripening) [47, 48]. The moment the external matrix reaches its gel point with respect to these aging processes determines the eventual pore size and interconnectivity [22, 49]. Systems based on gelatine, alginate, or other polysaccharides solidify under mild conditions and therefore naturally lend themselves to foam templating [50, 51]. However, achieving very small pore sizes (<100 µm) is challenging when relying solely on mechanical foaming, because the gelation must outpace drainage and Ostwald ripening‐driven coarsening to preserve the smallest bubbles, which are intrinsically unstable in polydisperse gas‐liquid foams [52, 53, 54]. HIPE templating extends the accessible chemical space by enabling hydrophobic continuous phases, such as monomeric or oligomeric precursors of synthetic polymers [55], that are difficult to accommodate in classical liquid foams where the dispersed phase (gas) is itself hydrophobic. By replacing gas bubbles with a densely packed liquid internal phase, HIPEs allow the formulation of reactive matrices that can be polymerized and/or crosslinked via a considerable number of strategies (e.g., traditional radical polymerization [56], controlled thiol‐ene “click chemistry” [57], ring‐opening polymerization [58], etc.) which significantly broadens the range of achievable compositions, mechanical properties, and post‐processing routes. Although foams and emulsions are often treated as separate communities, their physics becomes congruent once the internal‐phase fraction exceeds close‐packing (and emulsions become HIPEs). In fact, both bubbles and droplets adopt polyhedral contacts separated by thin films and Plateau borders, and the same balance of capillary pressure, viscous dissipation, and interfacial rheology governs their stability [59]. For example, this shared geometric and physical backbone means that the mechanism of window formation between adjacent pores (formerly, bubbles or droplets) during solidification is analogous across systems. In both liquid foams and HIPEs templating, interconnects originate from localized film‐thinning at bubble or droplet contacts, and this process is highly sensitive to surfactant distribution [21, 60]. In situ observations show that windows can nucleate from surfactant‐rich regions, zones that thin preferentially and may later be removed during curing or washing, which explains why surfactant type and concentration so strongly toggle between open‐ and closed‐cell outcomes [21, 61].

One of the practical distinctions between liquid foams and HIPEs is where the processing burden lies. HIPE templating requires stabilizing very high internal‐phase volumes and subsequently removing 74%–95% of a liquid template; hence, the access to a broader chemical palette these systems grant comes with important demands on surfactant content and purification protocols. On the other hand, foam templating relies on a more limited set of accessible chemistries but avoids the extraction of an internal liquid phase and is therefore operationally simpler. Due to their shared physics, bulk‐generated HIPEs and foams (bulk foaming and shearing) also share a fundamental limitation: because bubble and droplet size distributions are typically broad, each compartment follows a different drainage rate, capillary pressure, and film‐thinning mechanisms. Polydispersity drives local variations in how fast thin films evolve, how surfactant partitions at the interfaces, and when neighboring compartments come into contact or rupture [59, 62]. As a result, stabilization and destabilization pathways become strongly size‐dependent and spatially heterogeneous, hindering the direct correlation between the fluid template and the final porous solid.

If monodispersity of the internal phase (bubbles or droplets) is achieved, the translation from template size to pore size could more easily become one‐to‐one, since monodispersity minimizes the thermodynamic, capillary, and transport gradients that drive most destabilization mechanisms [63, 64]. Such systems make the architectural promise of fluid templating explicit but also highlight why the deterministic generation of compartments is the decisive stage of the process. This is the conceptual point at which microfluidics becomes consequential: by producing bubbles and droplets with narrow size distributions and by arranging them under confinement, microfluidic templating stabilizes the link between formulation and solid architecture and, crucially, enables on‐demand modulation of compartment properties before solidification. The next section briefly illustrates the micro‐hydrodynamics and interfacial rheology that enable this determinism and establishes the operational parameters that later translate into programmable gradients during printing.

### 2.3 Microfluidic Control of Bubble and Droplet Formation in Newtonian and Non‐Newtonian Fluids

The shift from bulk templating to microfluidic templating stems from a simple but transformative idea: when interfacial breakup occurs under confinement and the flow remains in the linear regime, bubble and droplet formation becomes deterministic [23]. Within microfluidic channels, inertia and gravity are negligible and breakup is governed almost entirely by the balance between viscous and capillary forces [65]. Under these conditions, the stochastic phenomena that dominate bulk emulsification and mechanical foaming are replaced by well‐defined hydrodynamic regimes (squeezing, dripping, and jetting) each of which links fluid properties and flow parameters to bubbles or droplets size with remarkable precision, making the production of foams and emulsions controllable and predictable. In the squeezing regime, typically accessed at low capillary numbers (C*_a_*, the ratio of viscous forces to interfacial tension forces), bubble and droplet size is set primarily by the channel geometry and the ratio of dispersed to continuous phase flow rates (*Q_d_*/Q*_c_)* [66]. Break‐up occurs when the growing inner thread blocks the channel and pressure builds until pinch‐off, a mechanism extensively characterized by Garstecki et al. [67] This regime is notably insensitive to interfacial tension forces, making it robust across diverse chemistries and surfactant systems. As flow rates increase, the system transitions to the dripping regime, where interfacial and viscous stresses balance to set a characteristic droplet length [68]; at even higher velocities or low interfacial tension, jetting regime emerges, enabling the generation of droplets whose size is determined by Plateau–Rayleigh instabilities downstream of a stable liquid thread (Figure 3) [69].

**FIGURE 3:**
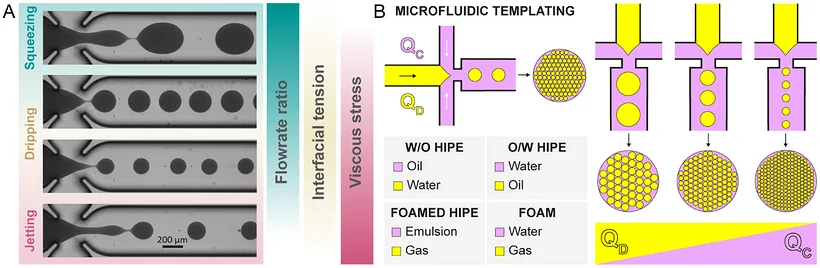
Deterministic control of bubble and droplet formation in confined microfluidic geometries. **A**: representative optical micrographs of a flow‐focusing junction showing the main interfacial breakup regimes: squeezing, dripping, and jetting. The colored gradient bars schematically indicate the relative contribution of the key parameters governing breakup, that are flow‐rate ratio, interfacial tension, and viscous stress. The color intensity reflects the relative weight of each contribution within a given regime. In the squeezing regime, breakup is mainly controlled by the balance between the dispersed and continuous phase flow rates, whereas interfacial effects become increasingly relevant in the dripping regime. At higher flow conditions, viscous stresses progressively dominate, leading to jetting. These different force balances result in characteristic and reproducible compartment sizes and inter‐droplet spacings. Reproduced with permission under the CC‐BY licence [69] 2019, The Authors. Microfluidics and Nanofluidics published by Springer Nature. **B**: microfluidic templating strategies based on controlled generation of droplets or bubbles upstream of manufacturing. Here, the balance between continuous‐phase and dispersed‐phase flow rates (Q_C_ and Q_D_) is explicitly used to prescribe compartment size and internal phase volume fraction, enabling the controlled formation of water‐in‐oil (W/O) an oil‐in‐water (O/W) high internal phase emulsions (HIPEs), foamed HIPEs, or liquid foams.

For the microfluidic generation of stable foams and emulsions, the use of a surfactant is inevitable and its influence on the process is not negligible. Indeed, surfactants play a dual role. Beyond simply lowering interfacial tension, their adsorption kinetics and interfacial rheology modulate film thickness, resistance to thread elongation, and the critical capillary number defining regime boundaries. In particular, slow‐adsorbing surfactants can generate transient Marangoni stresses that suppress necking, while strongly elastic interfaces alter pinch‐off times and stabilize smaller droplets [70]. Notably, the parameters that set droplet size also influence bubble packing and ordering downstream: under laminar flow, monodisperse compartments self‐organize into crystalline or pseudo‐crystalline lattices whose arrangement is dictated by geometry and interfacial energy rather than by buoyancy or turbulent collisions [71].

We underline, however, that much of the current understanding of microfluidic bubble and droplet generation has been established using low‐viscosity Newtonian liquids, for which breakup dynamics are primarily governed by the interplay between viscous and capillary forces. The translation of these concepts to the microfluidic templating of porous polymers is not straightforward, as the continuous phase must ultimately serve as the precursor of the solid final material. Consequently, it should consist of polymer solutions or pre‐polymeric mixtures (polymer/monomer viscous systems, resins etc.). These media often exhibit substantially higher viscosities than the model fluids commonly used in microfluidic studies and frequently display complex rheological responses, including shear‐thinning behavior and viscoelasticity. Under such conditions, the flow behavior of the continuous phase becomes central to bubble and droplet generation [72, 73], influencing not only the breakup dynamics but also the range of accessible operating regimes and, ultimately, the characteristics of the resulting template. In other words, rheology becomes a key design parameter. Indeed, experimental studies have demonstrated that the rheological properties of the continuous phase directly affect boundaries separating squeezing, dripping, and jetting regimes, modifying the operating window available for controlled bubble and droplet generation. In shear‐thinning fluids, for example, dripping can be observed under conditions where Newtonian fluids would still exhibit squeezing behavior [72]. Rheology also influences the processes of bubble/droplet neck formation and pinch‐off. Since bubble and droplet generation is a dynamic process (involving thread growth, interface deformation, neck thinning and eventual breakup), the timescale of each stage fundamentally controls the final outcome. Such timescales are directly related to the relative magnitudes of viscous, capillary, inertial and, when present, elastic stresses. Rheological properties therefore influence not only the final compartment size, but also breakup frequency, detachment position and filament stability [74]. The importance of rheology becomes even more evident when considering dimensionless groups commonly used to describe microfluidic generation of bubbles and droplets. Parameters such as the Capillary number, Weber number, and viscosity ratio all contain rheological information either explicitly or implicitly. Hence, any attempt to predict bubble or droplet formation in non‐Newtonian formulations requires an accurate description of the flow behavior of the continuous phase over the relevant range of deformation ranges [74].

## 3 Microfluidic Fluid Templating for the Fabrication of Polymeric FGPMs: From Concept Validation to 3D Printing Integration

Microfluidic generation of bubbles and droplets in crosslinkable and polymerizable fluids may be non‐trivial. However, this has not prevented researchers from exploiting the deterministic nature of microfluidics the fabrication of ordered porous polymers from fluid templates. This approach enables an exceptional degree of structural control, allowing the production of both highly monodisperse systems and architectures with precisely tailored gradients. The earliest demonstrations of fluid templating, where the fluid precursor was faithfully transferred into its solid counterpart, were developed independently of 3D printing technologies. Additive manufacturing was incorporated only later, once fluid templating strategies had matured sufficiently to satisfy kinetic and rheological requirements necessary for printability. The integration of 3D printing introduced an additional level of geometrical freedom, enabling enhanced spatial control over pore architecture and providing unprecedented opportunities for the on‐demand design of porosity. This section first surveys the most significant examples from the recent literature on microfluidic fluid templating of porous polymers; subsequently, we review the available studies combining microfluidic fluid templating with 3D printing for the fabrication of polymeric FGPMs.

### 3.1 Early Demonstrations of Microfluidic Control in Fluid Templating of Porous Polymers

Elsing et al. investigated monodisperse foamed emulsions (emulsions in which air is introduced as a “second” internal phase) and microfluidic HIPEs in which droplet or bubble size were directly translated into the porous structure of the solid polystyrene foams after polymerization, with window (pore interconnections) sizes tuned independently by surfactant concentration rather than by droplet size [75]. Costantini et al. extended this concept to dextran methacrylate‐based polyHIPEs, preserving FCC/HCP‐like packing over mesoscopic distances and further confirming that the interconnect diameter and the number of throats per pore could be predictably tuned by formulation [76]. These results highlight a crucial advantage of microfluidic templating: the independent control of size, packing, and interconnectivity, which is not achievable in bulk emulsification.

On the other hand, microfluidic foam generation follows similar principles but constrained by gas compressibility and the need to stabilize thin films more rapidly. Andrieux, Drenckhan, and Stubenrauch demonstrated that microfluidic flow‐focusing combined with controlled, time‐dependent gas‐pressure modulation enables the production of both monodisperse and polydisperse liquid foams [24], with independently tunable bubble size and polydispersity (but not pore gradients). Colosi et al. conducted a similar study on PVA‐based foams, also comparing pore‐size distribution obtained via mechanical and microfluidic foaming [77]. In alginate systems, Costantini et al. schematically mapped how gas fraction and surfactant concentration, at fixed bubble size, shape the number and size of interconnects between pores [78].

Eluding the classical definition of microfluidics but strongly entangled with its fundamental principles and applied to produce porous solids, it is important to mention a peculiar technique introduced by Visser et al. in 2018, called in‐air microfluidics [79]. This approach (which later provided the basis for “direct bubble writing (DBW)”, a method that integrates microfluidics, 3D printing, and graded porous polymers production, as discussed in Section 3) relies on the controlled collision of microscale liquid jets and droplet trains directly in air. Here, monodisperse droplets, generated via jet breakup, impact a second liquid jet and become “encapsulated” by it, effectively forming a kind of single‐droplet emulsion. The process is primarily governed by capillary forces and interfacial tension gradients (Marangoni effect), which promote coalescence while limiting droplet deformation and drive the encapsulation mechanism. Overall, the coupling of in‐flight impact, surface tension driven encapsulation, and (optional) rapid solidification of the outer shell enables the continuous, high‐throughput production of monodisperse droplets with tunable size and composition.

Understanding the physics of breakup and compartment organization in the discussed systems, led to the production of gradients as a natural extension of the method. Indeed, by varying gas pressure dynamically during foaming, Elsing et al. produced continuous or stepwise pore‐size gradients in polystyrene foams, with density gradients emerging under foamed‐emulsion templating conditions and pure pore‐size gradients emerging under HIPE conditions [80]. Practical considerations (e.g., viscosity‐limited gradient smoothing, the need for refined pressure ramps, the risk of weak interfaces at sharp transitions, etc.) were also articulated, illustrating the coupling between hydrodynamics and solidification. All these studies, summarized in Figure 4, showed that gradients can be written on demand simply by modulating flow or pressure inputs, provided that the continuous phase solidifies rapidly enough to capture the imposed structure.

**FIGURE 4:**
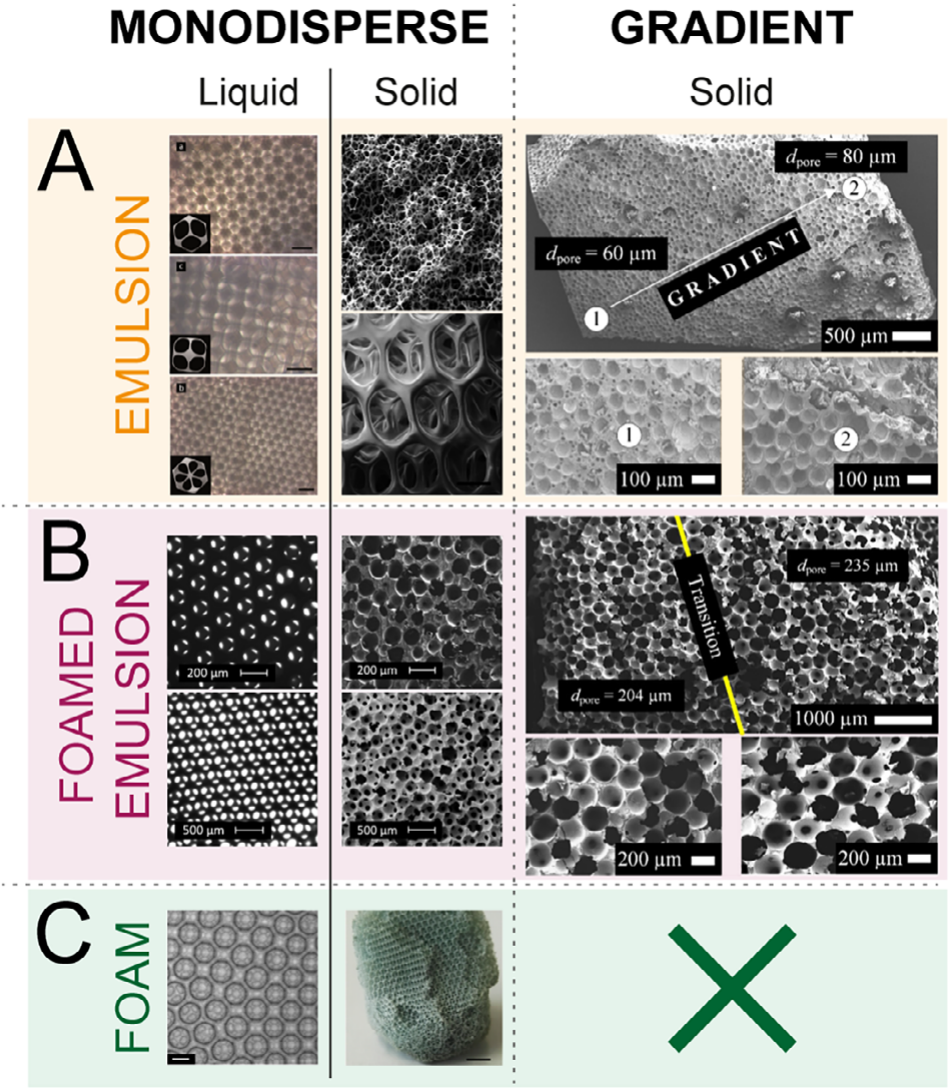
Representative porous architectures obtained from microfluidically‐generated fluid templates, arranged to compare three templating systems (rows: emulsions, foamed emulsions, and liquid foams) across two architectural outcomes (columns: monodisperse and spatially graded porosity). In the case of monodisperse templates, both the liquid (template) and the solid outcome (polymer) is shown. **A**: microfluidic emulsions are shown to yield highly ordered, monodisperse structures. Reproduced with permission [76] 2014, Royal Society of Chemistry. Microfluidic emulsions have been also used as templates to generate controlled spatial gradients in pore size. Reproduced with permission [80] 2017, Wiley‐VCH GmbH. **B**: microfluidic foamed emulsions combine emulsion templating with a gaseous internal phase, enabling high porosity, intermediate levels of ordering, and limited examples of graded microporous architectures. Foamed emulsion has been used as templates to produce solid polystyrene foams. Reproduced with permission [75] 2017, Royal Society of Chemistry. On the right, polystyrene foams with density gradient from foamed emulsion templating. Reproduced with permission [80] 2017, Wiley‐VCH GmbH. **C**: In contrast, although microfluidic liquid foams have been extensively employed to generate monodisperse porous structures, well‐defined pore size gradients have not been reported to date without the use of additional downstream structuring or printing steps. Reproduced with permission [24] 2018, American Chemical Society.

Thanks to microfluidics, foams and HIPEs evolved from stochastic dispersions to programmable liquid architectures, in which bubble or droplet size, spacing, composition, and volume fraction can be varied over time with exquisite precision. This determinism is the critical enabler for the next methodological leap: integrating bubble and droplet generation directly at the printhead allows to couple temporal control of the templating dispersion with spatial control during deposition. In what follows (Section 3.2), we give an overview of the emerging platforms that merge microfluidic control with additive manufacturing, enabling the fabrication of programmable polymeric FGPMs.

### 3.2 Microfluidic Fluid Templating Integrated in 3D Printing Platforms

Microfluidic templating provides exceptional control over bubble and droplet monodispersity, packing, and volume fraction. However, although order and monodispersity consistently mitigate destabilization phenomena in foams and HIPEs, these systems remain thermodynamically metastable, and instabilities will emerge (at rates slower than in their polydisperse counterparts) if the system is not ‘frozen’ within the appropriate time window. Once a templated dispersion leaves the microchannel, it still encounters drainage, coalescence, coarsening, and interfacial rearrangements, which occur on timescales comparable to those of deposition. The history of microfluidic‐assisted 3D printing is therefore best understood as a progression of strategies developed to reduce, manipulate, or eliminate the unstable temporal window between interfacial breakup and material solidification. A visual summary of said strategies is reported in Figure 5, and their outcomes (pore dimensions and distribution) as well as the employed materials are collected in Table 1.

**FIGURE 5:**
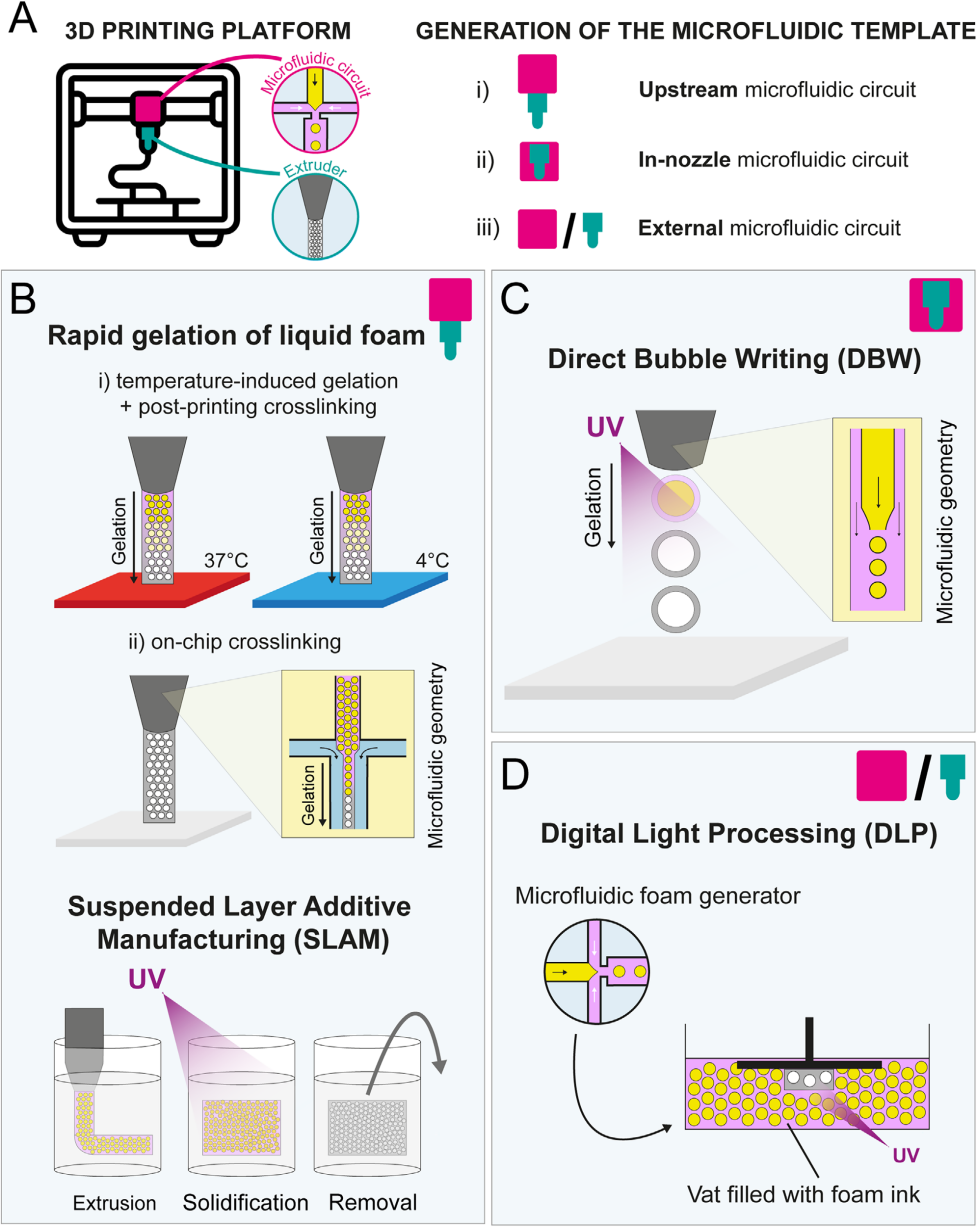
Overview of the microfluidic‐assisted additive manufacturing routes for producing polymeric functionally graded porous constructs from fluid templates. **A**: Main configurations of a microfluidic‐integrated 3D printing platform. The chip can be i) positioned upstream the printing head/nozzle, ii) integrated within the nozzle or iii) separated from the printing head/nozzle and used to fill a vat. **B**, **C**, and **D** present the strategies to stabilize and solidify the microfluidic‐generated template. In all schematics, the external (continuous) phase is shown in pink when in the liquid state and in grey after solidification, while the internal templating phase (droplets or bubbles) is shown in yellow or white (for appropriate colour contrast with respect to the external phase and with no relation to its state).

**TABLE 1:** Summary of the available studies on microfluidic templating integrated in 3D printing platforms for the production of polymeric FGPMs.

Author, Year	Template type	Microfluidic set‐up	3D printing set‐up	Material(s) (external phase)	Stabilization and solidification methods	Microfluidic‐generated bubble/droplet sizes	Pore distribution	Pore sizes (in porous solid)	Template/Solid fidelity
Costantini (2019) [81]	Foam	Valve‐Based Dynamic Flow‐Focusing (vFF)	Extrusion in Air	Gelatin	Thermogelation on Refrigerated Plate, Freeze‐Drying, Slow Crosslinking	80–800 µm	Continuous Gradient	80–800 µm	Overall maintenance of pore dimensional range, slight blurring of the gradient, pore volume faction increased by 10%.
Marcotulli (2023) [82]	Emulsion	Flow‐Focusing	Suspended Layer Manufacturing (SLAM)	DexMA, GelMA	Viscoelasticity of Suspending Bath + UV	100–400 µm	Alternate Layers, Step Gradient, Radial	N.D.	Pore distribution and gradients are spatially maintained. Volumetric porosity in solid decreases by 30%–40% with respect to fluid template.
Tirelli (2026) [83]	Emulsion	Mix‐Flow‐Focusing	Suspended Layer Manufacturing (SLAM)	DexMA, GelMA	Viscoelasticity of Suspending Bath + UV	100–400 µm	Continuous Gradient	100–230 µm	Gradient is maintained with 7 discriminable pore sizes populations.
Nalin (2023) [84]	Emulsion	Pressure‐Actuated Step‐Emulsification	Suspended Layer Manufacturing (SLAM)	Fibrinogen, DexMA, GelMa	Viscoelasticity of Suspending Bath + UV	65–550 µm	Continuous Gradient	N.D.	Overall maintenance of prescribed porosity profiles.
Weber (2024) [85]	Foam	Valve‐Based Dynamic Flow‐Focusing (vFF)	Digital Light Processing (DLP)	GelMA	Photopolymerization in Vat	143–747 µm	None in 3D Constructs, Continuous Gradients Demonstrated in 2D	150–450 µm	N.D.
Dai (2024) [86]	Foam	Co‐Axial Glass Capillaries	Extrusion in Air	Alginate/Pluronic F127	Thermogelation on Heated Plate, Calcium‐induced Gelation	300–750 µm	Stepwise porosity% gradient, Continuous dimensional gradient	320–500 and 410–640 µm	Very good dimensional fidelity.
Serpe (2025) [87]	Foam	Flow Focusing	Extrusion in Air	Alginate, Gelatin, Laponite	On‐chip Gelation (Calcium‐induced Gelation)	25–73 µm	Continuous Gradient	N.D.	Pore gradients and layers with different porosities are maintained without any blurring (printed strands are clearly distinguishable).
Visser (2019) [88]	Single Bubble	Core‐Shell Nozzle, In‐Air Microfluidics	Direct Bubble Writing (DBW)	Aqueous Acrylate Formulation	No Need for Stabilization (no Liquid Foam), In‐Flight Photopolymerization	N.A.	None, Switch Between Open and Closed Pores (oxygen sensitive chemistry)	400–700 µm	Volumetric shrinkage calculated on the printed object with respect to the CAD: xy −28%, z −28%.
Amato (2020) [89]	Single Bubble	Core‐Shell Nozzle, In‐Air Microfluidics	Direct Bubble Writing (DBW)	Viscous Surfactant‐Free Thiol‐ene ink	No Need for Stabilization (no Liquid Foam), In‐Flight Photopolymerization	N.A.	Alternate Layers	90–122 µm	Negligible shrinkage, as declared by the authors.
Rastogi (2025) [90]	Single Bubble	Core‐Shell Nozzle, In‐Air Microfluidics	Direct Bubble Writing (DBW)	PEGDA‐Based Inks	No Need for Stabilization (no Liquid Foam), In‐Flight Photopolymerization	N.A.	Alternate Layers of Different Densities	N.D.	Shrinkage declared, but not quantified: the authors are not interested in pores dimension but in different densities and topologies for acoustic applications.

*Note*: **Template Type** indicates Fluid biphasic system used as template to be translated in the porous solid; **Microfluidic Set‐Up** indicates Geometry of the channels in the microfluidic chip; **3D Printing Set‐Up** indicates Type of manufacturing strategy; **Stabilization and Solidification Methods** indicates Ensemble of the strategies employed to *i)* arrest or slow down the destabilization of the original fluid template prior to *ii)* permanent solidification of the external phase.

Two foundational approaches emerged almost simultaneously in 2019: the deterministic foam‐printing route introduced by Costantini and co‐workers [81], and the conceptually innovative DBW developed by Visser and colleagues [88]. Although chronologically parallel, these methods define the opposite ends of a conceptual arc that is later completed by suspending‐bath approaches (Figure 6) and, ultimately, by in‐nozzle gelation strategies that synchronize microfluidic breakup and template solidification (Figure 7).

**FIGURE 6:**
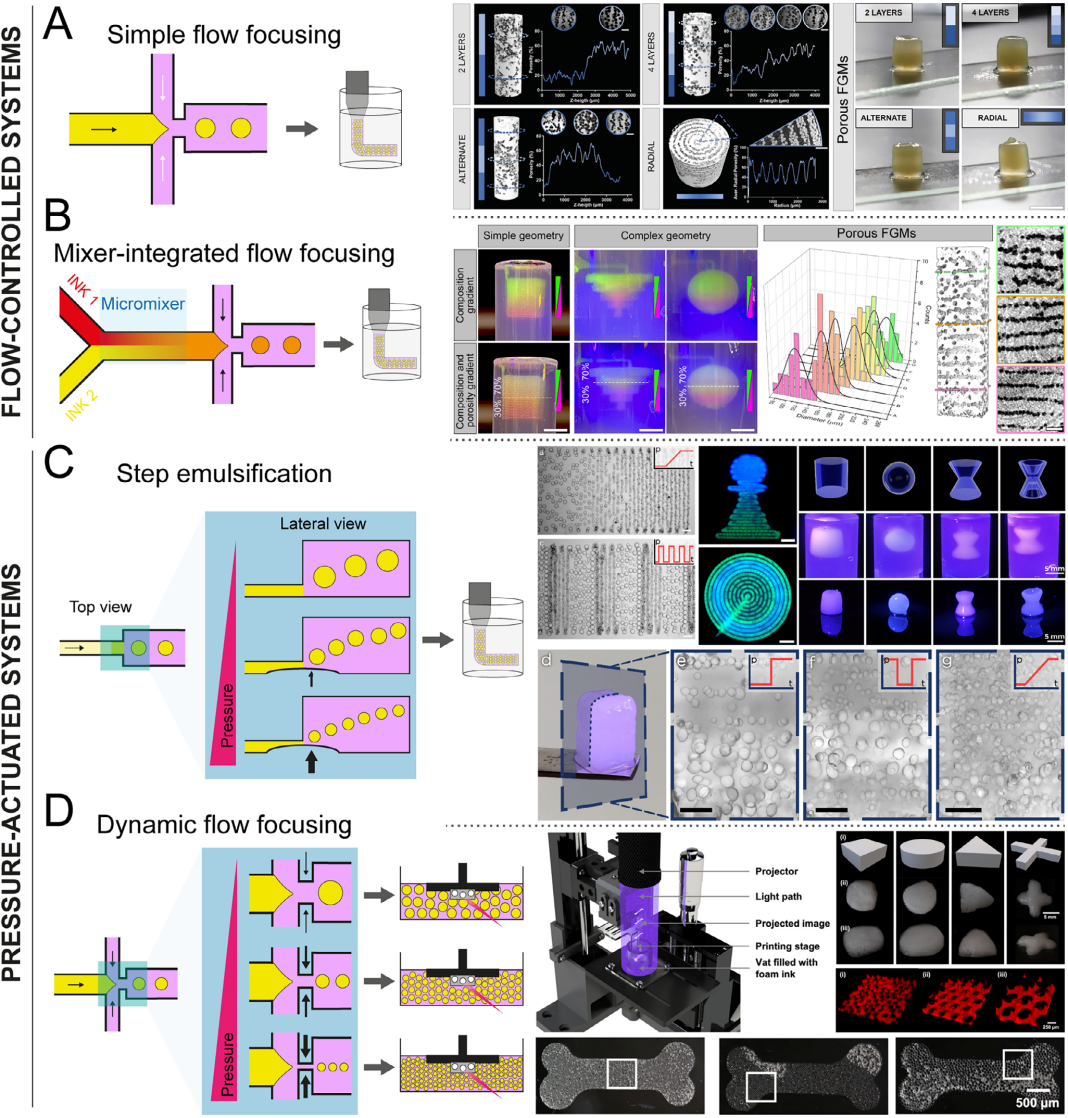
Bath‐based microfluidic‐assisted 3D printing systems for pFGPMs fabrication. For each literature example, a sketch of the microchannel geometry is provided on the left while images of the results on the right. **A**: Microfluidic‐enabled generation of FGPMs via emulsion templating using a simple flow focusing junction chip and a supporting bath. Micro‐CT scans, 3D reconstructions and photographs of printed FGPMs constructs generated with 2 and 4 layers, alternate and radial porosity distribution confirmed the obtainment of desired architectural geometries. Reproduced with permission [82] 2022, Wiley‐VCH GmbH. **B**: Mixer‐integrated microfluidic‐assisted 3D printing system for the generation of FGPMs with variable composition and porosity. Images of 3D‐printed constructs with simple and complex geometries with both compositional and architectural gradients and evaluation of the pore diameter via micro‐CT scans of the gradient porosity sample revealed the generation of a continuous gradient in porosity. Reproduced with permission [83] 2026, Royal Society of Chemistry. **C**: Microfluidic step emulsification to produce FGPMs via emulsion templating. Examples of 2D and 3D printing of variable porosities with alternate layers and gradient variation. Photographs of 3D‐printed constructs in the agarose bath: rendering of the STL file and 3D constructs before and after UV illumination and bath removal. Reproduced with permission under the CC‐BY licence [84] 2023, The Authors. Lab on chip published by Royal Society of Chemistry. **D**: Microfluidic‐based DLP printing system to produce porous constructs via foam templating. Rendering of the printing set‐up and examples of different printable shapes. Reproduced with permission under the CC‐BY licence [85] 2023, The Authors. Aggregate published by Wiley‐VCH GmbH.

**FIGURE 7:**
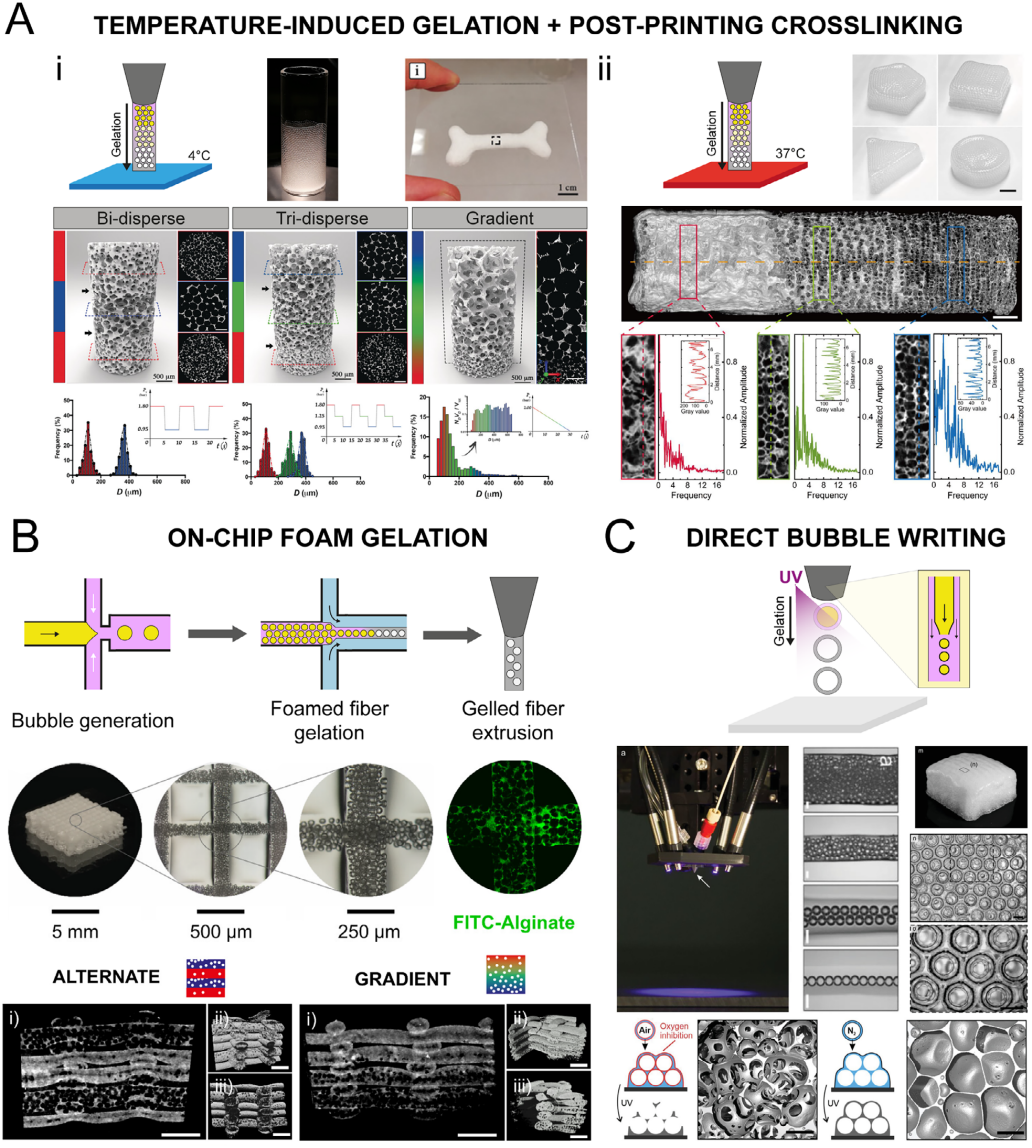
In air microfluidic‐assisted 3D printing systems for pFGPMs fabrication. **A**: 3D printing systems with temperature‐sensitive inks and post‐printing processing for the fabrication of tailored FGPMs. i) A cold plate is used to enable gelation of the gelatin‐based ink and subsequent processing to obtain chemical crosslinking. Formation of mono‐, bi‐, tri‐disperse and gradient porosity structures and analysis of the pore dimensions. Reproduced with permission [81] 2019, Wiley‐VCH GmbH. ii) A warm plate (37°C) guarantees the solidification of the extruded filaments once deposited. Subsequent treatments allow to stabilise the final structure and remove the internal component to generate pores. On top, images of the printed constructs. At bottom, micro‐CT scans confirmed the presence of an architectural gradient with pores ranging from 300 to 600 µm. Reproduced with permission [86] 2024, Elsevier Ltd. **B**: Microfluidic‐based 3D printing system for the fabrication of porous hydrogel with hierarchical arrangement of pores. On top, sketch of the microfluidic circuit, down an image of the 3D construct with successive closer view of the internal to highlight the presence of tiny gas bubbles. At bottom, micro‐CT scans of alternate and gradient porosity scaffold that confirmed the maintenance of the architectural design imposed. Reproduced with permission under the CC‐BY licence [87] 2025, The Authors. Published by Applied Materials and Interfaces by American Chemical Society. **C**: Direct bubble writing (DBW) to produce tailored polymeric foams. On the top, schematization of the physical principle behind DBW that allow single‐bubble ejection thanks to fast curing of the external shell. On the bottom, images of the printing set‐up, of foamed fibres at different gas fractions and of a 3D printed construct with close‐up. Finally, schematization of the process of polymerization of the fluid template to obtain open‐ and close‐cell geometries depending on the type of gas employed in the system. Reproduced with permission [88] 2019, Wiley‐VCH GmbH.

Costantini's demonstration can be considered the first clear example of what becomes possible in additive manufacturing when the mechanism of bubble formation itself is made programmable (Figure 7A, i) [81]. Their valve‐based flow‐focusing (vFF) microfluidic chip includes pneumatic control to allow reshaping of the breakup orifice in real time. Pressures between 0 and 2 bar altered the flow focusing junction width from roughly 120 µm down to about 20 µm, shifting the system between distinct breakup morphologies and enabling bubble diameters to vary by nearly an order of magnitude: from approximately 80 µm to over 800 µm, while achieving gas fractions up to 90%, and maintaining narrow size distributions (significantly narrower than those obtained by mechanical foaming). The challenge, however, lays in stabilizing a gelatin‐based foam whose viscosity is modest and whose drainage timescale at room temperature is far shorter than the duration of a typical print. Costantini's solution (printing onto a refrigerated plate to leverage the rapid sol‐gel transition of gelatin) allowed the foam to solidify fast enough to preserve monodispersity, producing the first printed porous construct with continuous pore‐size gradients spanning a full order of magnitude. Yet, the process required multistep solidification and was strictly limited to materials that gel upon cooling, thereby restricting the method's broader applicability.

The next generation of techniques shifted from arresting destabilization while being tied to a specific chemical composition, to slowing it down but accessing a larger material palette. Instead of altering gelation kinetics, researchers introduced yield stress or viscoelastic “suspending baths” (suspended layer additive manufacturing—SLAM), most commonly based on agarose fluid gels, which can support soft filaments prior to crosslinking while providing sufficient transparency for light penetration. Marcotulli and co‐workers demonstrated the power of this approach [82] using a microfluidic flow‐focusing printhead (ff‐MPH) that generated O/W emulsions with droplet diameters spanning roughly 100–400 µm and dispersed‐phase volume fractions (Φ) between 0.2 and 0.8 (Figure 6A). These emulsions, composed of dextran methacrylate (DexMA) or gelatin methacrylate (GelMA) continuous phases containing a surfactant and cyclohexane droplets, lack the elasticity necessary to retain shape in air. However, when extruded into the agarose fluid gel, each filament was mechanically supported until post‐printing UV crosslinking. The method enabled the fabrication of 3D constructs with alternating dense and porous regions, overhangs exceeding 45°, and radially modulated architectures. Nonetheless, droplet size remained largely fixed by the constraints of flow‐focusing, limiting the accessible pore‐size range to roughly a factor of two, and the suspending bath introduced diffusive smoothing that attenuated gradient sharpness.

By integrating a micromixer into the aforementioned system (Figure 6B), Tirelli and co‐workers [83] addressed one of the most challenging problems at the interface of biofabrication and materials science: fabricating constructs with simultaneous gradients in chemical composition and porosity. Their mixer‐integrated flow focusing printhead (mix‐ff‐MPH) incorporated a serpentine micromixer upstream of the droplet generator, where two aqueous polymer streams (one containing DexMA, the other GelMA) were folded and stretched into each other under chaotic advection before entering the flow‐focusing junction. The dispersed phase, hexadecane, formed droplets of 100–400 µm in diameter at dispersed phase fractions between 0.2 and 0.8. By modulating the ratio of the two polymer streams while maintaining constant total flow, Tirelli's system inscribed continuous transitions in matrix chemistry and local porosity simultaneously. Printed into an agarose bath at 35°C, the resulting filaments exhibited controlled transitions from DexMA‐rich to GelMA‐rich regions, as well as porosity gradients between approximately 30% and 70%. Yet again, the need for a suspending bath introduced temporal smoothing, and the droplet size remained constrained within a narrow operational window.

A more ambitious extension of the bath‐based paradigm arose with the development of dynamically tunable step emulsification. In microfluidic step emulsification (Figure 6C) the droplets are generated because of interfacial tension arising from a sharp geometric step in the microchannels rather than shear forces [91]. Nalin and colleagues [84] modified the classical configuration of step emulsification by introducing a pressure‐actuated membrane at the inlet of the emulsification chamber, enabling the orifice height to be altered during operation and granting access to droplet volumes spanning more than three orders of magnitude (Figure 6C). Their “tuna‐step” device produced both W/O and O/W emulsions thanks to surface‐modified PDMS channels made compatible with organic solvents, allowing long‐term production of droplets in systems containing fibrinogen, DexMA, or GelMA. Integrated with an agarose supporting bath, 3D constructs containing sharply alternating or smoothly varying porosity and even multimaterial patterns were obtained by rapidly switching continuous phase chemistries. Although remarkable for its scalability (including a 14‐nozzle parallelization), this approach still required a support bath and a purification step following UV‐curing, while membrane actuation introduced latency that smoothed gradients at high writing speeds.

Removing the suspending bath altogether was the natural next step, but doing so could expose a deeper mismatch between microfluidic timescales and the kinetics of photopolymerization. Weber and co‐workers attempted to circumvent this issue by directly feed microfluidically‐generated foams into a top‐down digital light processing (DLP) system (Figure 6D) [85]. In their design, bubble‐laden GelMA‐based resins with bubble diameters ranging from 143 to 747 µm were delivered into a DLP vat, where rapid layer‐by‐layer curing replaced the need for a support bath. Although this approach demonstrated that microfluidic foams could, in principle, be integrated with vat photopolymerization, it also revealed the intrinsic difficulty of controlling the foam‐air interface during layer formation. Indeed, surface tension produced non‐planar layers, bubble sizes limited resolution fundamentally, and high concentrations of photoinitiator induced excessive polymerization that resulted in blurred printed edges. A different “bath‐free” solution was proposed by Dai and co‐workers [86], who abandoned both suspending media and vat photopolymerization in favor of microbubble templating coupled with rapid thermogelation (Figure 7A, ii). Their coaxial microfluidic printhead generated monodisperse air bubbles (typically from 300 to 600 microns depending on the applied gas pressure) directly within a thermosensitive alginate/Pluronic F127 composite ink. In this case, the key was not instantaneous crosslinking, but rather a two‐stage solidification strategy: first, bubble‐laden filaments were deposited onto a heated (37°C) build plate, then the entire construct was immersed in a colder (20°C) CaCl_2_ bath to ionically crosslink alginate and leach out the Pluronic network. This decoupled stabilization allowed Dai's system to maintain fully bath‐free depositions while still achieving centimeter‐scale architectures with high pore monodispersity and tunable air fractions. Online modulation of air pressure and printing speed allowed to obtain both uniform and gradient macroporosity, preserving pore isotropy, despite the ink's softness, as confirmed by micro‐CT scans. While Dai's approach improves the resolution of the printed pFGPMs with respect to Weber's DLP method, it goes back to the reliance on thermogelation, re‐introducing a narrow thermal “printability window” and still requiring post‐printing treatment to stably hold the structure.

A fundamentally different insight arose when Serpe and colleagues reframed the problem not as one of supporting the foam, but of synchronizing bubble formation with crosslinking (Figure 7B) [87]. Instead of trying to preserve a metastable liquid dispersion long enough to print it, their two‐chip microfluidic printhead enabled foam solidification almost as soon as it was formed. In the upstream generator, a Laponite‐alginate‐gelatin solution containing a surfactant formed monodisperse bubbles between 25 and 73 µm, with polydispersity values often below 10%. In the downstream extruder, a second flow‐focusing junction introduced a CaCl_2_‐based crosslinking solution that initiated gelation within the nozzle. By minimizing the interval between breakup and solidification to the millisecond regime, Serpe's platform overcame the instability issues that had constrained all previous foaming approaches. The bubble‐filled filament exited the printhead already gelled, enabling deposition directly in air without thermal arrest or bath‐mediated stabilization. Gradients in porosity were inscribed simply by modulating input gas pressure, and micro‐CT reconstructions confirmed that both alternating and smooth gradients were preserved with high fidelity. Nevertheless, this approach is inherently limited by the use of rapidly crosslinking species (e.g., alginate), which inevitably constrains the material design space.

It is against this background that the work of Visser and co‐workers must be read. Although contemporaneous with the earliest demonstration by Costantini [81], their DBW approach provides the conceptual endpoint that the subsequent years of microfluidic printing techniques would converge toward gradually. Following an earlier demonstration of the method (originally referred to as “in‐air microfluidics”) [79], instead of printing/extruding a liquid foam that must be stabilized, Visser and coworkers eliminate the classical concept of foam altogether (Figure 7C) [88]. In DBW, a core‐shell nozzle emits liquid‐shell/gas‐core droplets at flow rates approaching 10 mL/min, and these droplets (typically 0.4–0.7 mm in diameter) are photopolymerized in flight and fully solidified within approximately 0.2 s after the impact on the collecting plane. By confining the existence of the bubble to a brief airborne interval, DBW bypasses the entire suite of foam‐aging phenomena. While the gas pressure modulates cell size and the relative density of the printed foam, the oxygen content of the core gas toggles between open‐ and closed‐cell structures, being the polymerization process oxygen‐sensitive. Because the key parameters governing breakup, shell thickness, and polymerization are all applied at the nozzle, DBW can write porosity at printing speeds up to 500 mm/s, far exceeding those of any microfluidic‐foam extrusion method presented, enabling high scalability. Nonetheless, the early DBW platform exposed two important constraints. The first was the limited operational window for stable bubble‐train ejection: indeed, outside a narrow band of gas pressures relative to the viscous and capillary counterpressure, the system transitioned from dripping to spray regimes with poorly defined bubble sizes. The second was shrinkage: the aqueous‐acrylate inks experienced roughly 28% contraction upon drying. Amato and colleagues overcame this limitation by developing a solvent‐free, surfactant‐free thiol‐ene ink [89] with viscosity near 410 cP (40 times higher than the earlier acrylate formulations) yet still compatible with stable bubble ejection at around 3 mL/min. This compositional refinement eliminated drying‐induced shrinkage and enabled the production of chemical gradients through stoichiometric imbalance of thiol and alkene groups, permitting targeted post‐functionalization of printed foams. By operating in a narrow pressure band (18‐22 kPa), the authors demonstrated transitions between nonporous strands, fine‐celled foams around 90 µm, and coarser foams around 122 µm without interrupting printing. A final challenge concerned the ballistic nature of bubble deposition. Because bubbles inherit the lateral velocity of the printhead during their free fall (typically around 10 cm), their landing position is offset from the nozzle trajectory. Rastogi and colleagues quantified this offset [92], showing that at speeds near 500 mm/s, radial deviations could reach up to 8 mm. By modelling bubble flight under negligible aerodynamic drag and applying toolpath compensation, they restored geometric fidelity for curvature features and sharp corners without sacrificing speed. More recently, Rastogi et al. also applied DBW to the fabrication of functionally graded acoustic solid foams [90], demonstrating that modulating the core‐gas pressure during flight‐polymerization can encode both density gradients and controlled open‐ and closed‐cell morphologies. Using PEGDA‐based inks, they produced centimeter‐scale sandwich foams with tunable stiffness and porosity, achieving sound transmission loss (STL) peaks up to 68 dB and broadband absorption down to 400 Hz, an example of DBW being repurposed not merely for geometric control but for tailoring a specific macroscopic function (i.e., acoustic dissipation).

What emerges, looking across these platforms, is not a sense of arrival but of acceleration. In only a few years, microfluidic breakup has evolved into a programmable actuation knob; foams and emulsions stabilization has migrated from exogenous supports to intrinsically synchronized kinetics. Consequently, porosity has shifted from an emergent property to a variable that can be imparted, modulated, and inscribed along a path. Each innovation step exposed a new constraint (i.e., limited pressure windows, actuation latency, diffusive smoothing in suspending media, ballistic offsets at high speed) and each constraint, once made explicit, became something that could be engineered around. That logic has not exhausted itself. What becomes clear when looking across these methods is not that the path forward is predetermined, but that the field is only beginning to discover the kinds of control it can exert. Each platform highlights a different piece of the landscape: the sensitivity of droplet and bubble generation to pressure transients, how filaments respond to acceleration or deceleration during deposition, the influence of dead volume and mixing length on gradient sharpness, or the narrow actuation windows that separate stable trains from dripping or spray. These observations suggest directions rather than prescriptions. Faster or more responsive actuation could help preserve gradient fidelity during changes in print speed, alternative nozzle geometries might widen the range of bubble sizes available without compromising monodispersity, and curing strategies that combine rapid initial arrest with slower consolidation could strengthen interfaces in sharply graded regions. None of these possible research paths are guaranteed, but all are now visible because the field has progressed far enough to make its bottlenecks legible.

## 4 Toward Programmable Porosity: Coordinated Control Across Formulation, Flow, and Architecture

Although microfluidic‐controlled 3D printing has begun to show how fluid templating can be exploited to impose porosity with spatial and temporal precision, the experimental palette supporting these demonstrations remains narrow. Most reported systems rely on alginate [86, 87], GelMA [82, 83, 84, 85], DexMA [82, 83, 84], one example of PEGDA [88] and a single case of a modern thiol‐ene click formulation [89], materials chosen for their familiarity and photoreactivity, rather than for the breadth of physical behaviors that functionally graded porous polymers could, in principle, access. The field has therefore developed its early proof‐of‐concepts within a rather tight corridor: mostly hydrophilic, water‐soluble precursors, UV‐initiated crosslinking, and viscosities easily compatible with smooth, laminar breakup. While entirely sensible from a scientific standpoint, this restriction leaves large regions of chemical and mechanical space untouched: fast‐curing resins, multiphase inks, compositions with delayed or multi‐stage gelation/solidification [93, 94], or chemistries that could support far more resilient or multifunctional polymeric FGPMs. Expanding this material space is not trivial. For example, shear‐thinning (non‐Newtonian) formulations, which are highly desirable in the extrusion printing field, are by definition characterized by a viscosity that depends on the applied shear rate. In a microfluidic setup, this breaks the linear correspondence between the imposed and experienced flow rates, making access to a desired break‐up regime unpredictable in the absence of extensive rheological characterization of the formulation. Highly viscous, resin‐like formulations are incompatible with current standard device designs, as the microfluidic channels would rapidly develop very high pressures, blocking the flow and challenging the pumping systems. Consequently, studying the correlation between viscosity thresholds and the minimum microfluidic bubble or droplet dimensions attainable in larger channels would be necessary to define the accessible workspace. Multiphase reactive systems could provide a smart strategy for accommodating different chemistries within tailored complex fluids; however, their reaction kinetics must be precisely mapped to demonstrate compatibility with both the millisecond timescales of microfluidic bubbling and the much longer timescales associated with 3D printing.

What we therefore envision is that the next generation of materials will not simply enter the experimental set‐ups demonstrated so far, but will challenge the rheology, the breakup physics, and the printing process simultaneously. These domains are highly interdependent: for example, a formulation chosen for its (final) mechanical or chemical features may destabilize breakup, while a microfluidic geometry optimized for one rheological regime may become inappropriate once the formulation is varied to impart desired viscoelastic features that ensure higher printing quality. In such settings, progressing by trial‐and‐error or by isolated experiments becomes impractical. What is needed are tools that can follow the material from its formulation through its processing (foaming/emulsification, extrusion, deposition,) and curing history, anticipate how each stage influences the next, and adapt predictions as the process evolves.

While constitutive rheological models [95], droplet‐generation theories [96], and computation fluid dynamics (CFD) simulations [97] provide important predictive capabilities at individual stages of the process, they are typically developed for specific physical regimes and become increasingly difficult to extend when rheology, interfacial dynamics, reaction kinetics, and printing conditions evolve simultaneously. In microfluidic‐controlled printing of polymeric FGPMs, formulation, breakup dynamics, extrusion behavior, and curing history are strongly coupled. Yet, a unified physics‐based predictive framework remains elusive. Data‐driven approaches and artificial neural networks (ANN) therefore offer a complementary strategy, capable of identifying nonlinear and history‐dependent relationships across the entire processing chain without requiring an explicit mechanistic description of every intermediate phenomenon.

In what follows, to contextualize the opportunities offered by ANN, we briefly examine the current state of ANN development across the domains relevant to microfluidic templating of 3D‐printed pFGPMs, highlighting both the progress achieved so far and the perspectives for their eventual integration into a unified framework.

At the rheological level, Mishra et al. have already shown that dynamic neural networks (such as NARX—nonlinear autoregressive model with exogenous input) can reconstruct the intertwined processes of structural breakdown, recovery, yielding, and viscoelasticity of a sample from a single transient test, outperforming classical structural‐kinetics models [98]. Their model goes further: once trained, it predicts an entire suite of rheological behaviors (hysteresis, creep, nonlinear oscillations, even apparent steady‐state flow curves) without any explicit constitutive equations. In practice, this means learning the material's time‐dependent features directly from a single or a few simple experiments, a capability with obvious implications for ink transport, print‐path stability, and bubble or droplet entrainment.

Other approaches (e.g., rheology‐informed and physics‐informed neural networks) embed the constitutive equations inside the learning process, recovering IKH (Isotropic Kinematic Hardening, an elasto‐visco‐plastic constitutive model) parameters from minimal data and making reliable predictions under flow histories that were never part of training, including start‐ups (sudden flow imposition) and oscillatory cycles [99, 100]. Such systems behave as proper “rheology twins” (i.e., neural‐network surrogates that behave like a virtual rheometer, trained on a small set of experiments) and their importance can become essential for designing inks with fast reactivity, for example. Indeed, in systems where viscoelasticity or polymerization kinetics can evolve during printing, they could offer a way to track the ink's operative window in real time, rather than relying on static rheometer values measured under idealized conditions.

The potential of neural networks is also evident on the microfluidic side. In this context, the advantage of using neural networks is the prediction of bubble sizes across different flow regimes and devices, resulting in excellent design ability. For example, Chang et al. demonstrated that a hybrid mechanistic‐data neural network trained on a multi‐study dataset can predict bubble size within a few percent error across T‐junction geometries and can be rapidly adapted to newly fabricated chips through transfer learning, requiring only a few calibration points [101]. Rahimi et al. even extended this logic to step‐emulsification, famously geometry sensitive and for long considered too multiparametric for compact and accurate predictions [102]. Here, a relatively small ANN trained on high‐fidelity 3D simulations could predict droplet diameter and generation rate with striking accuracy (R^2^ of 0.989 and 0.972, respectively) and identify the dominant geometric and fluidic factors. This demonstrates that machine learning surrogates can process information on geometrical asymmetry, interfacial dynamics, and multiphase interactions simultaneously, providing rapid design guidance in regimes where simple scaling laws fail. The need for tighter co‐design across formulation, microfluidic operation, and architectural outcome is pushing the field to evolve unusually fast. A recent review by Lai et al. reflects this acceleration and surveys the rapid expansion of AI‐assisted droplet microfluidics [103], from droplet generation and detection to adaptive control and multimodal data integration, underscoring how quickly these capabilities are maturing and diversifying.

Finally, once the templated architecture forms, graph neural networks (GNNs) offer a language for translating pore geometry into functional predictions quickly enough to be potentially taken into consideration already during fabrication, allowing for the adjustment in real time of the processing parameters. As an example, pore‐GNN frameworks trained on micro‐CTs achieve permeability and formation factor (a parameter linked to pore connectivity) predictions with R^2^ values in the 0.90–0.95 range while remaining computationally lightweight, and graph‐adapted Grad‐CAM provides interpretable pore‐level insights that would be difficult to extract from volumetric GNNs. These are demonstrated primarily on geological samples, but the methodology is not system‐bound and completely transferable. At a more structural scale, GNN surrogates trained on dynamic simulations of impact, bending, and buckling predict full‐field strains at 7–15x the speed of particle methods (e.g., Material Point Method–MPM, or Discrete Element Method–DEM) while maintaining physically meaningful accuracy, exactly the kind of capability that will matter as polymeric FGPMs evolve toward mechanical functions beyond simple cushioning. Mesh‐to‐graph approaches (consisting in converting physical simulation data, typically represented as a mesh, into a graph structure to be analyzed via GNN), as in the work of Maurizi et al., show that wrinkling, post‐buckling, and localized deformation can be learned from a few hundred finite‐element exemplars and then evaluated in subseconds, opening the door to real‐time structure‐property feedback as materials and architecture diversify [104].

Seen in this light, the role of neural networks is not to replace established physical understanding but to mediate between processes that, until now, have been explored separately because the materials were simple enough to allow it. As chemistry expands, the dependencies between rheology, microfluidic breakup, and final properties will tighten. The most realistic path forward therefore lies in developing co‐registered data streams and interoperable neural modules: a “rheology twin” trained on operational histories, a microfluidic predictor‐controller that shares state with it, and a graph‐based surrogate that reads newly formed architectures and returns property estimates fast enough to guide the next steps in the print (a simplified example of what an integrated ANN framework could look like is proposed in Figure 8). This is not yet the reality of the field, but the individual components already exist, and their integration is less a conceptual hurdle than an engineering one. As these connections are built, functionally graded porous polymeric materials may shift from being a clever demonstration of paired technologies into a genuinely programmable class of engineered matter, where pore architecture can be tailored to simultaneously optimize transport [105], mechanical [106], acoustic [107], or biological performance [108] depending on the target application [109].

**FIGURE 8:**
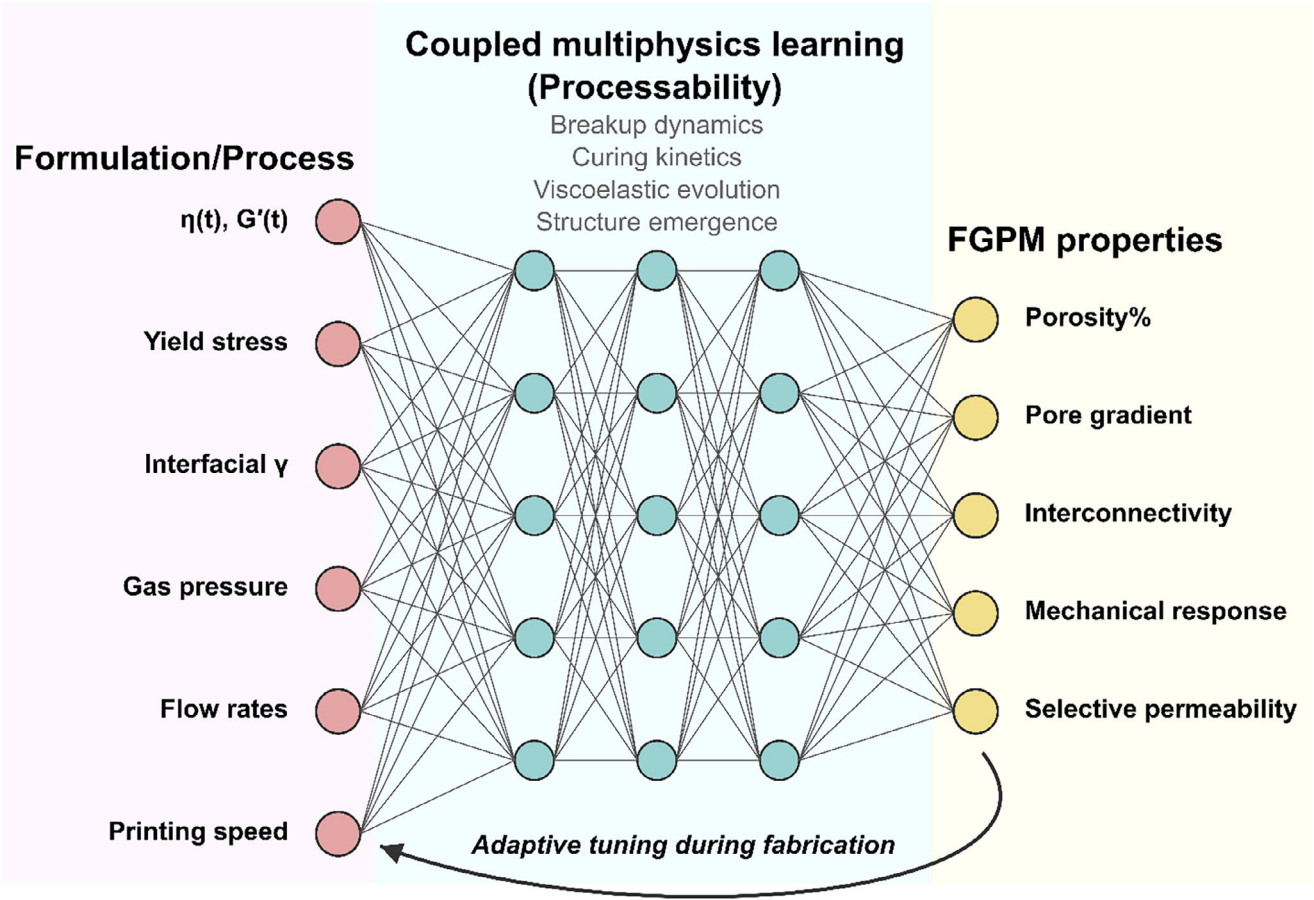
Conceptual representation of a neural‐network (NN)‐assisted framework for the fabrication of polymeric functionally graded porous materials (pFGPMs) via microfluidic templating integrated in additive manufacturing platforms. Measured and controllable formulation and process variables (left; e.g., time‐dependent rheology, interfacial properties, gas pressure, flow rates, printing speeds) are provided as inputs to a neural network. Hidden layers represent the coupled learning space in which multiphysics interactions (such as emulsion or foam breakup dynamics, curing kinetics, viscoelastic evolution and structure emergence) are captured without explicit mechanistic decoupling. Network outputs correspond to architectural descriptors and functional responses of FGPMs (right), including overall porosity, pore‐size gradients, interconnectivity, mechanical response, and transport‐related properties. The curved feedback arrow indicates adaptive tuning during fabrication. The scheme is intended as a conceptual, idealized framework illustrating the potential of coordinated, data‐assisted control of graded porosity.

## 5 Conclusions

pFGPMs can, in principle, offer a strikingly broad range of performance. Their controlled fabrication, however, remains nontrivial. In recent years, fluid templating of gellable, crosslinkable, and polymerizable liquid foams and HIPEs has started to reshape approaches to control the final microarchitecture of these materials. A decisive step forward came with the introduction of microfluidics, which enables active control over the size and polydispersity of the internal phases that ultimately template the void architecture. By restoring a degree of determinism in bubble and droplet production within biphasic systems, microfluidics provides not only improved microarchitectural control but also slows the onset of foam and HIPEs aging processes, extending the temporal window available for solidification and thereby improving the fidelity with which the fluid template is translated into the final porous solid. By integrating microfluidics into the printhead of additive manufacturing platforms, fluid templating can be translated into pFGPMs with precise control over pore size, distribution, and gradients, while maintaining the macroscopic design freedom of 3D printing. However, the field remains at an early stage and is only beginning to reveal both its potential and its limitations. One prominent constraint is the relatively narrow range of explored chemical systems, which currently relies predominantly on water‐soluble polymers and precursors compatible with laminar flow and smooth breakup conditions. While these systems have been necessary in establishing the foundation of the field, further progress will require expanding the accessible chemical space toward more robust and multifunctional materials suitable for real‐world applications. Looking forward, the key challenge lies in the strong interdependence between chemistry, formulation, stability, processability, printability, curing, and the resulting morphology‐property relationships. Progress in this direction will likely require addressing these aspects in a coordinated manner rather than isolation. In this context, a co‐design paradigm emerges as a natural evolution of the field, potentially supported by the rapid development of ANN, which are already demonstrating considerable promise in areas such as microfluidics, rheology, and structure prediction in porous systems. The remaining challenge is their integration into interoperable workflows capable, in principle, of guiding the development of new formulations by simultaneously accounting for rheology, microfluidic processability, printability, and final performances. Achieving this level of integration could shift the fabrication of pFGPMs from a largely laboratory‐driven activity toward a scalable and genuinely programmable class of advanced materials.

## Funding

Vanessa Rosciardi acknowledges financial support from the Italian Ministry of University and Research (MUR) under the National Recovery and Resilience Plan (PNRR), Mission 4, Component 2, Investment 1.2, Young Researchers, project PRR.AP015.142 – PRR_M4C2_GR, BiFuelleD, CUP B83C25001210005. Andrea Barbetta acknowledges financial support from Sapienza University of Rome, Ateneo 2024 funds, N°RM12419112CDC234.

## Conflicts of Interest

The authors declare no conflicts of interest.

## Data Availability

Data sharing not applicable to this article as no datasets were generated or analyzed during the current study.
